# Genetic dynamics in the sand fly (Diptera: Psychodidae) nuclear and mitochondrial genotypes: evidence for vector adaptation at the border of Iran with Iraq

**DOI:** 10.1186/s13071-016-1603-5

**Published:** 2016-06-03

**Authors:** Sahar Ebrahimi, Ali Bordbar, Parviz Parvizi

**Affiliations:** Molecular Systematics Laboratory, Parasitology Department, Pasteur Institute of Iran, Tehran, Iran; Microbiology Research Center (MRC), Pasteur Institute of Iran, Tehran, Iran

**Keywords:** *Cyt b* gene, *EF-1α* gene, Genetic adaptation, Iran-Iraq boundaries, Landscape fragmentation, Molecular analyses, Phlebotominae

## Abstract

**Background:**

Our investigation uses nucleotide variations of the genera *Phlebotomus* and *Sergentomyia* using the *EF-1α* and *Cyt b* genotype regions to describe the sand fly fauna and genetic aspects collected at war-torn sites of the Khuzestan boundary between Iraq and Iran.

**Methods:**

All sand fly species were characterized using molecular genetics. The field work was conducted in six districts including 24 locations in remote areas for three years at the peak of sand fly activity during cutaneous leishmaniasis (CL) transmission seasons. The distribution of CL vectors was determined based on the climatic regionalization using the kriging method in ArcGIS model. DNA of sand fly pools were screened via polymerase chain reaction (PCR) using neutrality (Tajima’s *D*) and neutral allele frequency (Fu’s *F*_s_) tests to measure the effect of randomly evolving DNA sequence on the genetic diversity of sand fly populations in response to habitat fragmentation and landscape modification.

**Results:**

Among the 1213 specimens, ten species were identified based on morphology. The non-native species *Phlebotomus sergenti* was unequivocally found for the first time in the studied regions. Nucleotide substitutions of sand fly sequences varied most in the most disrupted districts (Dashte-Azadegan and Abadan; disparity index test: *P* < 0.05). The haplotypes of *Cyt b* from the subgenus *Sergentomyia* and *P. papatasi* revealed more heterogeneity (Tajima’s *D* > +2) than *P. alexandri* (*D* > +1), which suggests widespread heteroplasmic mitochondrial DNA mutations in the same mtDNA gene among different sand fly species. Subgenus  *Sintonius* exhibited greater fitness (*D* = 0) and (neutrality test; *P* > 0.05) no evidence of selection. The sequence of the nuclear gene *EF-1α* indicated similar nucleotide differences, as observed for the *Cyt b* gene, in all sand fly species, but lower levels of polymorphisms (*D* > +1) were observed compared with the mitochondrial *Cyt b* gene (*D* > +2) in the subgenus  *Sergentomyia*.

**Conclusion:**

Our findings describe random nucleotide diversity in the *Phlebotomus* and *Sergentomyia* population gene pools due to recent anthropogenic influence. A phylogenetic analysis showed that the closely related species are positioned in monophyletic clades, except for the subgenus *Sergentomyia* and *P. sergenti*, and highlights the importance of haplotype variations for the development of adaptability.

## Background

Hematophagous phlebotomine sand fly females are medically important but only some are proven leishmaniasis vectors with critical vectorial capacity and competency [1]. Sand flies transmit *Leishmania* spp. through their geographical distribution due to landscape fragmentation, migration, environmental and climatic oscillations. Certain fundamental criteria support the notion that sand flies are potential vectors: the capacity of sand flies to use their midgut receptor to attach a specific *Leishmania* species, behavioral or genetic diversification, vector-parasite coevolution and the adaptive manipulation of their interactions [2–5].

In a noteworthy Zoonotic Cutaneous Leishmaniasis (ZCL) focus, in a tropical climate region in southwestern Iran, Khuzestan Province has suffered from the Iraq-Iran war (from 22 September 1980 to 20 August 1988) and has recently been involved in Daesh terrorist destruction, which has led to ecological instability in the region, providing adequate conditions for flourishing heterogeneity and allowing for great diversity among sand fly species. The ZCL incidence was low during war time, but it suddenly soared among soldiers with over 10,000 annual cases reported from the public health centers of Khuzestan [6]. The prevalence range 1.8–37.9 % and incidence rate of 69,000–113,300 CL cases were reported by the Iran Ministry of Health and Medical Education and as WHO estimates, respectively [7, 8]. Despite the large and diverse sand fly distribution range in the geographical borders of the Khuzestan Province between Iran and Iraq, the sand fly species and the population structure of CL sand flies are not well-known. The commemoration of a religious man, Imam Hussein, is one of the largest religious gatherings in the world and a large number of Iranian and/or Afghan people (about two million pilgrims annually) walk through CL areas from across the Iranian boundaries (Khuzestan and Ilam Provinces) to Iraq. Indigenous and traveler populations are at risk for *Leishmania* infection by different sand fly species in many active transmission areas between expatriates and visitors. Simultaneous utilization of the sequence of applying both mitochondrial *Cytochrome b* (*Cyt b*) and conserved nuclear marker *elongation factor-1α* (*EF-1α*) as proper DNA markers have already provided data for analyzing the phylogenetic relationship among sand fly species [9]. These genes provide sufficient information to discern the species’ ancestor population in the natural habitat of phlebotomine sand flies [10].

The maternally-inherited mitochondrial *Cyt b* gene, reflects the evolutionary history because of high evolution rate, lack of recombination and clonal inheritance [11, 12]. The *Cyt b* gene has also been used for analyzing the genetic divergence within and among phlebotomine sand fly populations, mainly due to its high variability [1, 13, 14].

The nuclear gene, *EF-1α*, has a remarkable character to detect heterozygosity, haplotype diversity and genetic variation. The *EF-1α* gene has been identified as a potentially useful gene for studies of high-level phylogenetic relationships, particularly in insects [10, 15]. Many reports consider *EF-1α* as a proper marker for methodological and analytical challenges in systematic biology and molecular phylogeny [16–18].

In this study, we investigate species of the genus *Phlebotomus* as the main vectors of leishmaniasis as well as species of the genus *Sergentomyia* in studied areas. It is noticeable that DNA of *L. major* and *L. tropica* was definitely identified from *Sergentomyia* spp. by PCR-RFLP and ITS1 gene sequencing in Ghana and West Africa [19]. Initial morphological identification of sand fly species followed by molecular confirmation allowed us to improve effective, targeted and control measurements. To our knowledge, although some combined data from the markers of mitochondrial genes (e.g. *Cyt b*, *Cytochrome c oxidase subunit I* and *NADH4*) and/or nuclear genes (e.g. *EF-1α* and ribosomal DNA) [10, 12, 20] have achieved accurate information for the sand fly identification and population genetics, the neutrality test of loci has rarely been investigated [21]. Whereas sand flies pose substantial human health concerns, the objectives of this study were to identify collected sand flies to the species level using morphological and molecular characteristics and to determine the sand flies’ evolutionary relationships with landscape fragmentation using neutrality and phylogenetic analyses along the *c*.420 km Khuzestan borders.

## Methods

### Origin, collection and morphological identification

**Fig. 1:**
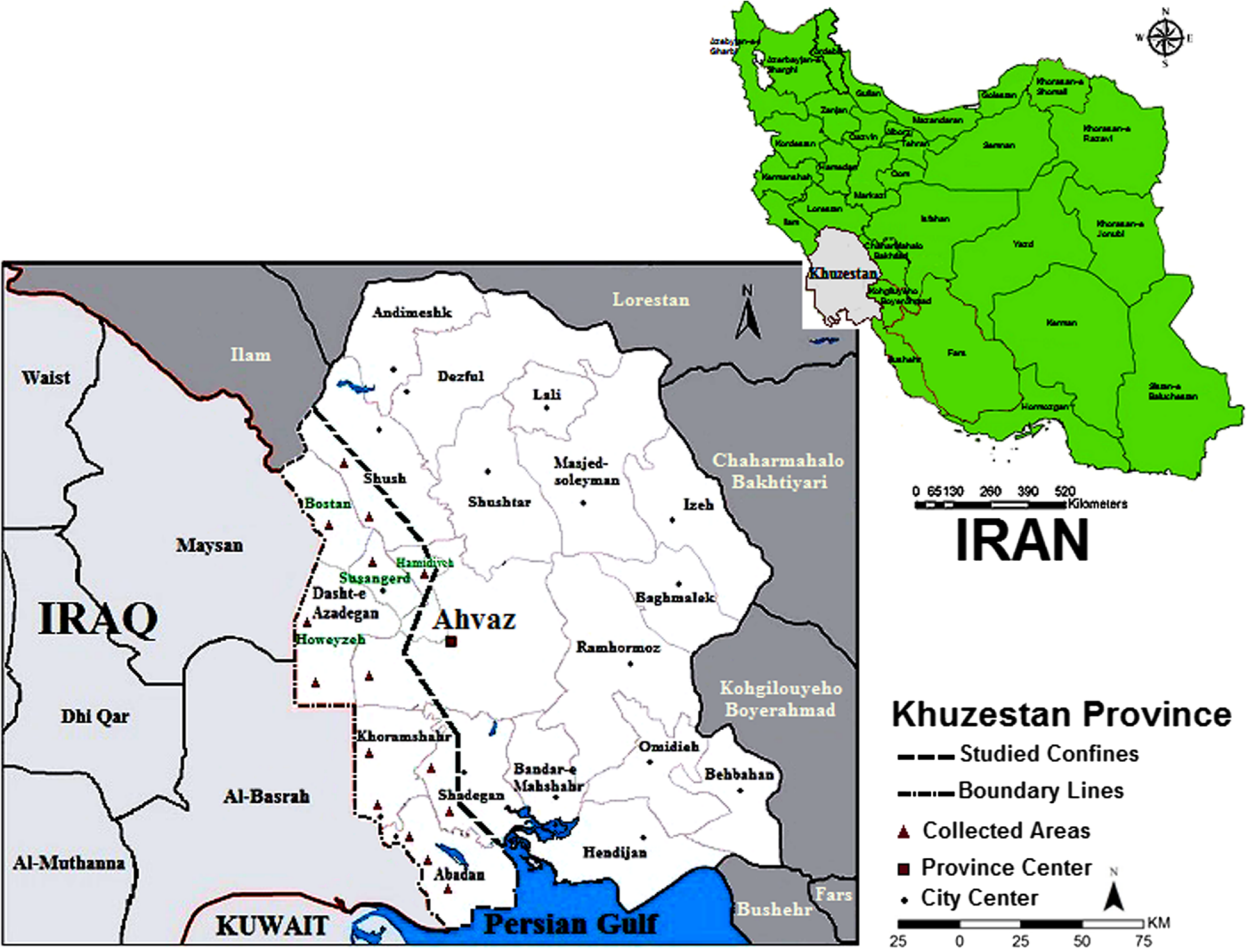
Map of Khuzestan Province of Iran with the collection sites for the sand flies caught across the boundary lines with Iraq (the main districts are confined within the dashed outline)

Sand fly species were caught on a large geographical scale in Khuzestan Province, which is located southwest of Iran (30°20′21″N to 31°33′29″N and 48°18′15″E to 48°10′51″E), bordering with Basrah and Maysan, Iraq provinces in the west and the Persian Gulf in the south (Fig. 1). The climate changes and type of weather in the studied areas differ from the other CL foci conditioned in Iran, with humidity ranging from 50 to 85 %, temperature ranging from 20 to 60 °C and a monthly average precipitation between 17 to 25 mm. Sand fly specimens were collected from 24 sites adjacent to Iraq’s borders in western parts of Khuzestan within approximately 10,406 km^2^ during their seasonal activity in late May to late November 2011–2014 (Fig. 1). The deterministic spatial interpolation was performed based on ArcGIS model using kriging method and bioclimatic variables obtained from Ebrahimi et al. data [22]. The climatic regionalization was used with the principal component analysis (PCA) and the clustering integration method (CIM) to obtain the appropriate classification.

Sticky sheets of paper and CDC miniature light traps were placed 1–1.5 m above the ground to sample the sand flies and were deployed overnight (before dusk until dawn). Sand fly traps were placed in a natural field of destroyed war remnants, natural and artificial holes and crevices, agricultural fields, gerbil burrow entrances, new developing urban areas, rural areas adjacent to the boundary lines, domestic animal shelters and around houses close to gerbil burrows two times per month (5 days each time) [23, 24]. All sand flies were examined and identified based on morphological characteristics of the head and abdominal terminalia using compound microscopy (400×). The genitalia of each sand fly were carefully removed using micro-needles and slides-mounted in Berlese's fluid following dissection using sterilized forceps [24].

### DNA extraction and PCR amplification of the *Cyt b* and *EF-1α* gene regions

Because a certain level of ambiguity and/or similarity appeared among female sand fly morphological characteristics of closely related species; the collected sand flies were molecularly characterized using the *Cyt b* and *EF-1α* gene regions. PCR fragments were amplified as previously described [10, 11, 14, 25]. The total DNA of the dissected thorax and anterior abdomen of individual sand flies was extracted using the modified method of Ish-Horowicz [26], GeNet Bio and a DynaBio™ Kit (Bioneer corporation, Seoul, Korea and Takapouzist corporation, Tehran, Iran). One hundred nano grams of each purified DNA sample was cycle-sequenced using an *AccuPower*® DNA Sequencing Kit from Bioneer and 3730xl/Bioneer 3730xl sequencing systems with 3.2 pmol of the same primers used for PCR. Nucleotide sequence data and new identified haplotypes of *Phlebotomus* and *Sergentomyia* spp. reported in this paper are submitted to the GenBank, EMBL, and DDBJ databases under accession numbers KX024717-KX024728; KX067785-KX067788 and KX088453-KX088456 (*Cyt b* gene) and KX099722-KX099736 (*EF-1α* gene).

### Molecular genetics and phylogenetic data analyses

After sequencing the PCR amplicons (both strands), all DNA sequences were exported to Sequencher™ v.4.1.4 for PC (Gene Codes Corporation) to identify the limits of the open reading frames (ORFs). The primers were trimmed from the sequences, aligned based on deduced amino acid sequences and visually edited, which yielded sequences between 758–790 nucleotides in length for the *Cyt b* gene region and 430–497 for the *EF-1α* including the primers. Visual analyses were used to assess heterogeneity and/or single nucleotide polymorphisms (SNPs) in both directions. A disparity index test for substitution pattern homogeneity was calculated with 500 Monte Carlo replications within *P. papatasi*, *P. alexandri* and *Sergentomyia* spp. populations (*P* < 0.05) [27].

The phylogenies were reconstructed to evaluate the species status and establish the relationships from the common and new haplotypes of the *EF-1α* and *Cyt b* Phlebotominae sand fly sequences. A maximum likelihood (ML) analysis using a heuristic search through stepwise addition of 100 random replicates and bootstrapping with 1000 replicates were performed using the MEGA 5.05 package based on the Tamura-Nei model of a nucleotide substitution search from Clustal W [28]. To group the various sand fly species, the phylogenetic maximum likelihood (PhyML) [29] program was run to obtain a maximum likelihood tree, and nonparametric ML bootstrap was used with 1000 replicates.

To test the differences in sand fly species abundance, statistical analyses were performed on all of the *Phlebotomus* species individually as main vectors susceptible to maintaining the transmission cycle of *Leishmania* parasites in the region and *Sergentomyia* spp., to be non-borne disease vectors. The MEGA 5.05 program was also used to calculate the number of variable nucleotide sites, nucleotide diversity (average number of nucleotide differences per site between any two randomly selected sequences) and the transition/transversion ratio (*R*). The potential selection pressure of the protein-coding sequences was determined using the dN/dS ratio test and a Z-test for selection based on the Nei-Gojobori method. DnaSp 5.10.01 was used to evaluate haplotype diversity (HD) and to analyze the value of Fu’s *F*_s_ (neutrality and allele frequency) for each species [30].

## Results

### Sand fly collection, morphological identification and sequenced specimens

**Fig. 2:**
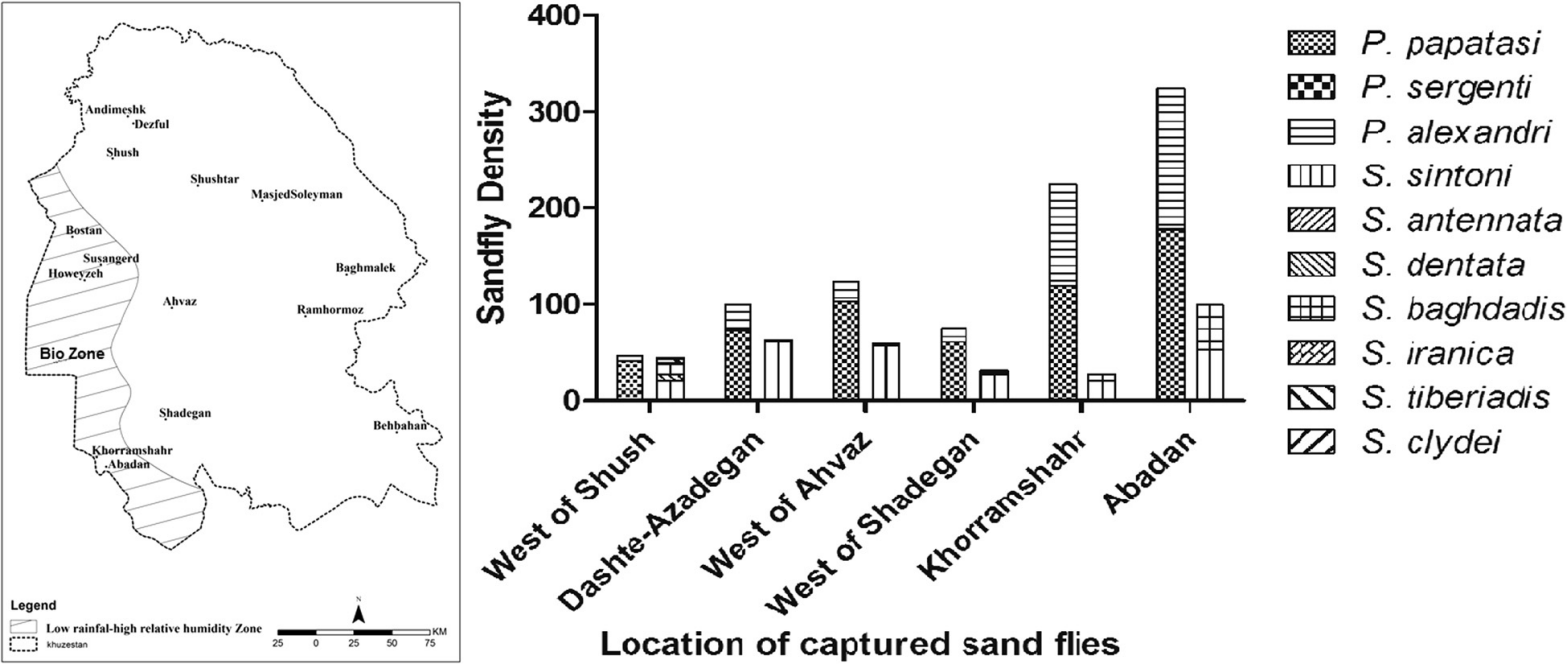
Bioclimatic regionalization of Khuzestan borders generated by the kriging method using principal component analysis (PCA) and the density of various collected sand fly species in low rainfall and high relative humidity bioclimatic zone

For this investigation, we identified ten species among *Phlebotomus* and *Sergentomyia* sand flies caught in 23 villages and a riverside next to the Khuzestan boundaries (Table 1). Three species of the genus * Phlebotomus* (*P. papatasi*, *P. alexandri* and *P. sergenti)* and seven species of the genus *Sergentomyia *(*S. sintoni*, *S. antennata*, *S. dentata*, *S. baghdadis*, *S. iranica*, *S. clydei* and *S. tiberiadis*) were found among a total of 1213 sand flies, including 893 *Phlebotomus* and 320 *Sergentomyia* specimens (Table 1). The studied area was identified as the low rainfall and high relative humidity zone on the basis of the effects of principal factors on climatic variables obtained from Ebrahimi et al. [22] (Fig. 2). We also successfully collected *P. sergenti* in the Dashte-Azadegan district and Abadan for the first time (Table 1).

**Table 1:** Fauna and distribution of sandflies collected from six districts along the Khuzestan boundaries, characterized based on morphological feature examinations and molecular analyses (M: male, F: female; data on sandflies that were not collected are not shown in this table)

				*Phlebotomus*					Sequenced genes		*Sergentomyia*													Sequenced genes		Total sandflies
Collection site				*Phlebotomus*		*Paraphlebotomus*					*Sergentomyia*					*Parrotomyia*		*Parvidens*		*Sintonius*						
				*P. papatasi*		*P. sergenti*	*P. alexandri*				*S sintoni.*		*S. antennata*		*S. dentata*	*S. baghdadis*		*S. iranica*		*S. tiberiadis*		*S. clydei*				
District	Location	Villages	Altitude (m.a.s.l)	M	F	M	M	F	*Cyt b*	*EF-1α*	M	F	M	F	F	M	F	M	F	M	F	M	F	*Cyt b*	*EF-1α*	
Shush	West	Haft tappeh	43–60	8	10		1	2	3	3	6	4			1	6	4			1	1			2	2	44
		Hanush		11	13		1	1			4	7						1	1		1	1	1			42
Dashte-Azadegan	Bostan	Mihan Abad	10	11	15		10	9	9	9	16	17							1					8	8	79
	Susangerd	Susangerd		9	12		3	1			8	9														42
	Howeyzeh	BaniAmeh		14	12	1	2	1			7	5														41
Ahvaz	North/ South-west	Hamidieh	9	10	16		5	3	4	4	5	6												3	3	45
		Ahvaz-Abadan road	10	12	16		6	5			16	6				2										63
		Gheyzaniyeh kuchak	7–9	14	10		1	0			5	5														35
		Seyedhasan	8	15	10		0	0			5	9														39
Shadegan	West	Darkhovin	6	10	13		2	3	3	3	4	6			1									3	3	40
		Mashmuli	9–10	10	8		2	1			3	5														29
		Shadegan	6–10	11	9		3	3			3	6	1	1	1								1			39
Khorramshahr	Eastern Houmeh	Sheneh	6.6	13	10		8	11	11	8	2	3				1	6							8	7	54
		Marzi	6.4	14	11		10	9			2	3														49
		Hanishieh	7.3	13	6		9	6			1	2														37
	Western Houmeh	Arayez	7	17	12		12	16			1	1														59
		Maslavi	6.8	11	12		13	11			4	2														53
Abadan	Nasar	Rofaye	5	15	8	1	11	12	13	9	12	16												11	8	75
	Northern Bahmanshir	Radeh Madan	3–5	14	9		10	8			3	1				3	1									49
		Kherkhereh	3–6	21	12		11	7			4	2														57
		AlbuEbadi	5–6	12	11		9	7			2	3														44
	Southern Bahmanshir	Radeh Sadat	3–5	14	13		14	12			1	1														55
		Abushanak	4–5	9	11		4	3			2	1				2	1									33
	Riverside	Arvandroud	1–2	16	12		22	16			3	2				23	16									110
Total (%)				575 (64.39)		2 (0.22)	316 (35.39)		43	36	241 (75.31)		2 (0.625)		3 (0.94)	65 (20.31)		3 (0.94)		3 (0.94)		3 (0.94)		35	31	1213
				893							320

Of 145 sequenced specimens for the *Cyt b* (78) and *EF-1α* (67) gene regions, 108 sequences could be analyzed for *Phlebotomus* spp. (*n* = 59) including *P. papatasi* (*n* = 16) and *P. alexandri* (*n* = 15) for *Cyt b*; *P. papatasi* (*n* = 15) and *P. alexandri* (*n* = 11) for *EF-1α* and two sequences of *P. sergenti* (*n* = 2) for both gene regions; and a total of 49 sequences from *Sergentomyia* specimens including *S. antennata* (*n* = 2) and (*n* = 3) for *S. dentata*, *S. tiberiadis*, *S. clydei*, *S. iranica* and *S. baghdadis* for both *Cyt b* and *EF-1α* gene regions, and *S. sintoni* (*n* = 11 and *n* = 10) for the *Cyt b* and *EF-1α* markers, respectively (Table 1). Altogether, 37 sequenced specimens of *P. papatasi* (*n* = 20) and *P. alexandri* (*n* = 17) did not have enough DNA or were not interpretable after resequencing.

### Molecular and statistical analyses of the *Cyt b* gene region

Our trimmed sequences, along with those deposited in GenBank, were analyzed to compare and identify new haplotypes (Tables 2, 3). After aligning and pruning all of the sequences, the total lengths of the *Phlebotomus* and *Sergentomyia* species were contained within 632–790 base pairs. The species *P. sergenti* and *S. iranica* were not statistically analyzed due to the limited number of sand flies caught in the studied areas (Table 4). Further, a single species, *S. baghdadis* (KH300), which does not transmit disease, was not statistically analyzed. The sequenced region of the *Cyt b* gene was A-T rich in all species (Table 4).

**Table 2:** All variant haplotypes of the *Cyt b* gene and indicated SNPs in sandflies showing inheritance of one or other parental allele isolated from Iran-Iraq boundaries (Unamplified sequences and identities in relevant positions are denoted by dashes and dots, respectively; M: male; F: female)

Species	Origin	Sex	Haplotype (GenBank acc. no.)	Position of nucleotide substitution																				
				1	1	3	5	7	7	7														
				7	9	4	6	3	4	4														
				8	8	2	7	2	3	4														
*P. papatasi*	Abadan (Italy)	M	KH382/535 (HM992927/HM992926)	G	T	C	A	C	A	T														
	Abadan	F	KH417 (KX024717)	A	.	.	.	.	.	.														
	Khorramshahr	F	KH419 (KX024719)	G	C	.	G	T	C	.														
	Abadan	M	KH421 (KX024718)	.	T	.	A	C	A	C														
		M	KH486 (KX024720)	.	.	T	G	.	.	T														
*P. alexandri*	O	S	H					2	2	3	5	5	5	6	6	6								
				3	4	5	8	0	6	0	0	5	5	2	4	7								
				9	5	4	2	8	3	4	8	4	9	8	6	9								
	Abadan	F	KH384 (KX024721)	A	T	T	G	T	A	A	T	T	G	A	T	C								
		M	KH528 (KX024725)	T	A	C	A	C	G	G	A	C	A	G	A	T								
	O	S	H		2	2	4	5	7	7	7	7	7	7	7									
				4	1	2	1	2	0	2	2	4	4	5	6									
				5	1	3	2	7	9	2	4	4	5	4	0									
	Germany		(HM803186)	-	-	-	-	A	T	T	A	A	T	G	G									
	Bostan	M	KH407 (KX024722)	T	T	T	T	C	.	.	.	.	.	.	.									
		F	KH410 (KX024724)	A	.	.	.	.	.	.	.	.	C	A	A									
	Howeyzeh	M	KH408 (KX024723)	.	G	C	C	A	C	C	T	C	.	.	.									
*S. sintoni*	O	S	H		2	2	3	6																
				3	7	8	0	0																
				3	9	0	7	1																
	Shadegan	M	KH133 (KX067785)	G	G	G	T	T																
		F	KH134 (KX067786)	A	.	.	.	.																
	Bostan	M	KH322 (KX067787)	.	A	A	C	C																
		F	KH327 (KX067788)	.	.	.	T	T																
*S. antennata*	O	S	H			2	2	4	6	6	6	6												
				5	8	2	3	7	3	7	7	8												
				4	4	2	1	7	0	0	5	0												
	Shadegan	M	KH108 (KX099719)	A	T	G	C	C	T	T	C	T												
		F	KH154 (KX099720)	T	C	A	T	T	C	C	T	A												
*S. iranica*	O	S	H					1	1	2	2	3	3	3	3	3	3	4	4	5	5	5	5	6
				4	7	7	8	0	1	5	7	0	2	3	3	5	8	1	2	1	1	7	9	2
				2	2	6	8	8	1	2	3	1	7	0	1	8	1	7	6	0	9	9	7	7
	Susangerd	F	KH135 (KX024727)	G	C	C	T	A	A	T	T	G	A	A	T	C	C	G	G	T	C	C	G	C
	Bostan	M	KH519 (KX024728)	A	T	T	C	G	T	C	C	A	T	T	C	T	T	A	A	C	T	T	A	T
*S. tiberiadis*	O	S	H	1	1	3	6																	
				0	5	6	9																	
				8	0	1	4																	
	Haft tappeh	F	KH305 (KX088454)	G	C	T	T																	
	Hanush	F	KH307 (KX088455)	A	T	C	C

**Table 3:** Variable haplotypes found in the alignment of species of *Phlebotomus* and *Sergentomyia* based on molecular analyses of the *EF-1α* nuclear gene obtained from Iran-Iraq boundaries

Gene	Species	Origin	Haplotype (GenBank acc. no.)	Position of nucleotide substitution																			
*EF-1α*	*P. papatasi*					1	1	2	2	2	3	3	3	3	4	4	4	4	4	4	4	4	4
				3	7	0	6	0	2	7	3	8	8	9	0	0	1	1	2	2	2	3	3
				9	8	8	2	4	2	0	9	6	7	5	7	9	5	9	0	2	4	0	2
		Abadan	KH382 (KX099735)	T	C	C	A	A	T	T	C	T	C	C	A	G	C	A	G	T	T	A	G
			KH421 (KX099736)	G	T	A	G	G	C	C	T	G	A	A	T	A	G	T	C	A	G	T	A
	*P. alexandri*	O	H	2	2	2	2	3	3	3	3												
				4	4	9	9	0	1	5	8												
				3	9	1	7	6	8	5	1												
		Abadan	KH384 (KX099733)	A	G	T	G	T	T	C	A												
		Howeyzeh	KH408 (KX099734)	G	A	A	C	C	A	T	G												
	*S. sintoni*	O	H			2	4	4	4	4	4												
				3	4	9	4	5	6	6	7												
				6	2	7	7	9	2	8	1												
		Shadegan	KH133 (KX099722)	C	A	T	T	C	G	T	A												
		Bostan	KH322 (KX099723)	.	.	.	C	.	.	.	.												
		Shadegan	KH134 (KX099724)	G	C	G	.	A	C	G	C												
		Bostan	KH327 (KX099725)	.	A	.	.	.	.	.	.												
	*S. tiberiadis*	O	H		2	3	3																
				5	9	1	9																
				7	4	5	0																
		Haft tappeh	KH305 (KX099726)	T	C	C	G																
		Hanush	KH307 (KX099727)	C	T	T	A

**Table 4:** Tajima’s *D* neutrality analyses and neutral allele frequency test (Fu’s *F*_s_) of the *Cyt b* and *EF-1α* gene regions in *Phlebotomus* and *Sergentomyia* species

Species	*P. papatasi*	*P. alexandri*	Subgenus *Sergentomyia*	Subgenus *Sintonius*	
Genes	*Cyt b*	*Cyt b*	*Cyt b*	*EF-1α*	*Cyt b*
Fragment length (bp)	788	789	789	497	789
*m*	6	5	7	6	6
*S*	431	428	367	276	35
*p*_s_	0.555	0.560	0.682	0.584	0.0445
*Θ*	0.243	0.269	0.278	0.256	0.0296
*π*	0.332	0.335	0.375	0.305	0.0296
*D*	2.391	1.876	2.041	1.250	0
A-T ratio (%)	70.92	73.84	68.4	46.82	70.02
G-C ratio (%)	29.08	26.16	31.6	53.18	29.98
*G*	3.632	7.265	1.11	2	0.05
*K*_*1*_	13.762	2.88	54.065	12.325	13.039
*K*_*2*_	9.879	3.198	60.416	1.391	20.427
Fu’s *F*_s_	3.047	3.568	*Sergentomyia* genus		-0.463
			0.913	1.482	

*m* = number of sequences, *S* = number of segregating sites, *p*_s_ = *S*/*m*, *Θ* = *p*_s_/a_1_, *π* = nucleotide diversity, *D* is the Tajima statistic test, *G* = the discrete Gamma distribution, *K*_*1*_ and *K*_*2*_ are the transition/transversion bias rate ratios of purines and pyrimidines respectively

**Fig. 3:**
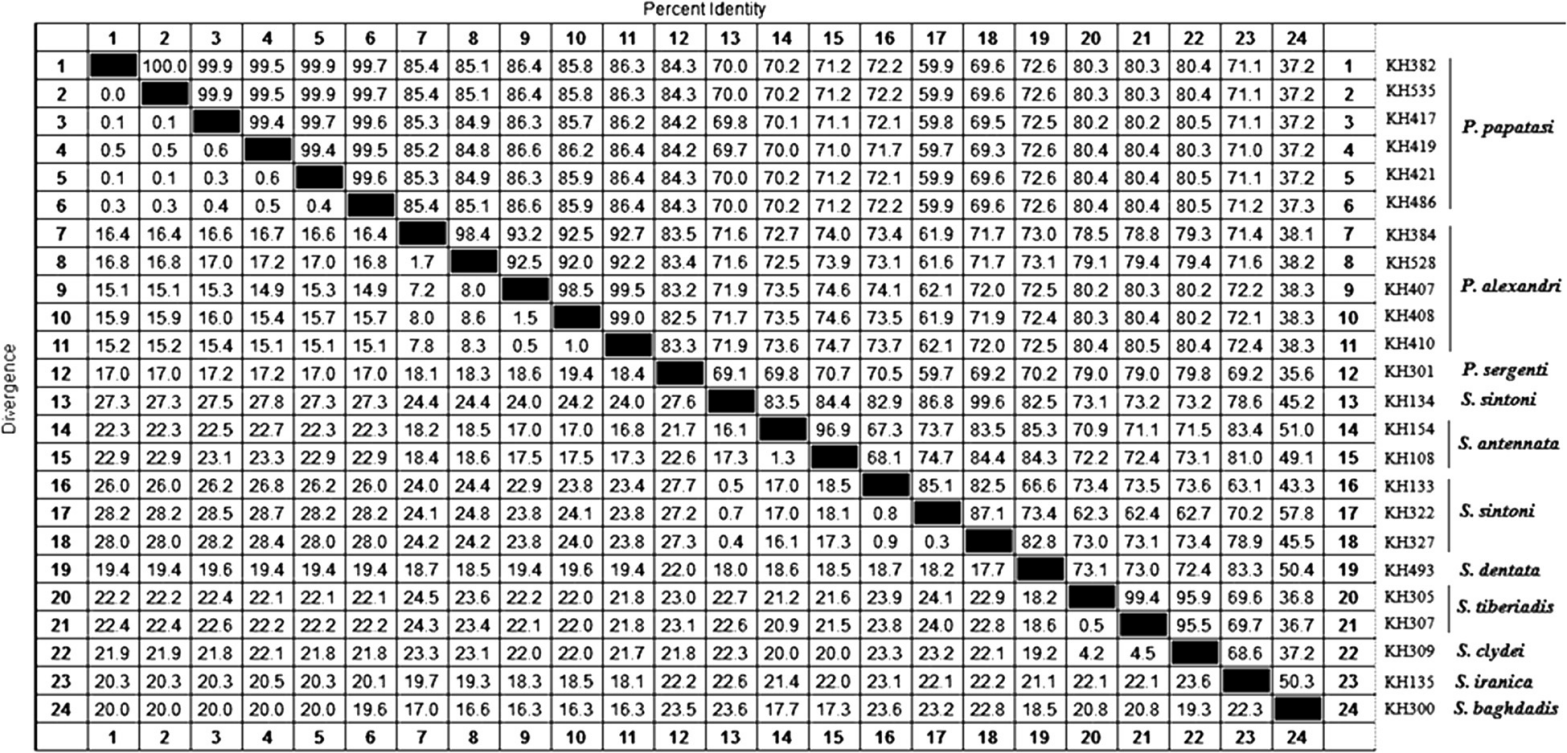
Pairwise comparisons of the nucleotide sequences among the species of *Phlebotomus* and *Sergentomyia* for the *Cyt b* gene region based on the percent divergence (below diagonal) and identity (above diagonal) in the studied CL foci

*Phlebotomus papatasi* had four unique haplotypes (KH417, KH419, KH421 and KH486) and a common haplotype of KH382, KH535 and other remaining sequences that had a 100 % similarity compared to haplotypes from Italy (GenBank accession nos. HM992926 and HM992927) (Table 2). Edited *P. alexandri* sequences revealed five unique haplotypes in two similar groups isolated from Abadan (KH384 and KH528) and from Dashte-Azadegan (KH407, KH408 and KH410) (Table 2). The haplotype diversity (HD) for genus *Sergentomyia* was *P =* 0.987 and three species of the subgenus *Sergentomyia* were characterized molecularly which showed four, two and one unique haplotype/s for *S. sintoni*, *S. antennata* and *S. dentata,* respectively (Table 2). In the subgenus *Sintonius*, three haplotypes were observed for the two species, *S. tiberiadis* and *S. clydei* (Table 2). Additionally, two new haplotypes were obtained for *S. iranica* (KH519 and KH135), with three A-T transversions and 18 transitions in the sequences (Table 2). The nucleotide diversity within the haplotypes of *P. papatasi* in Khorramshahr and Abadan and the subgenus *Sergentomyia* in all villages was generally significant (Tajima’s *D* > +2). The results of the neutrality test for *P. alexandri* haplotypes were considerable (*D* > +1, *P* = 1.876884) but lower than that for *P. papatasi* (*D* > +2, *P* = 2.391599), which indicates that the *P. alexandri* population is attempting to undergo equilibrium among gene frequencies and balancing selection (Table 4). The percentage of similarities and divergences among populations of  *Phlebotomus* and *Sergentomyia* are indicated in Fig. 3.

A statistical analysis of the selection pressures between the *P. papatasi* sequences clearly shows that the dN/dS ratios were strongly biased toward synonymous mutations (the dN/dS ranged from *P =* 0.008 of KH486/KH419 to *P =* 0.346 of KH486/KH417) in a total of 215 positions in the final dataset. This phenomenon is likely due to counterselection of deleterious mutations during the *Cyt b* gene evolution. Additionally, the neutrality test results were not considerable (*P* > 0.05) between the *Sintonius* spp. sequences.

### Molecular and statistical analyses of the *EF-1α* gene region

The total length of the *EF-1α* gene region was 497 bp for all species. The *EF-1α* gene exhibits higher GC content than the *Cyt b* gene in the subgenus  *Sergentomyia* (Table 4). The haplotypes identified from the *EF-1α* gene for each sand fly species take the following order: *P. papatasi, P. alexandri* and *S. tiberiadis* (two), and *S. sintoni* (four) haplotypes. Additionally, *S. antennata*, *S. dentata*, *S. baghdadis* and *S. clydei* each featured a unique sequence. Moreover, the KH421 sequence from *P. papatasi* exhibited 13 nucleotide differences compared with a previously detected sequence from the villages of Isfahan and Golestan areas (less affected regions by war), Iran (IRN377, GenBank accession no. EF416843), and *S. sintoni* haplotypes KH (133, 134, 322 and 327) exhibited 15 differences compared with the Iranian haplotype (IRN341, GenBank accession no. EF416846). Sequences for  *S. iranica* (KH519 and KH135) were not interpretable after resequencing.

Homogeneity was examined based on whether differences in base composition bias between sequences within the subgenus  *Sergentomyia* (Disparity index test) were significant (*P* < 0.05); significant differences were only observed between the KH108 (*S. antennata*) and KH134 (*S. sintoni*) sequences (*P* = 0.016). The remaining sequence estimates were equal (*P* = 1.000). The Z-test for selection pressure using the variance bootstrap method (dN/dS) was remarkable (*P* < 0.05) for closely related species of KH108/KH133, KH322 (*P* = 0.046, *S. antennata* and *S. sintoni*); KH108/KH327 (*P* = 0.020, *S. antennata* and *S. sintoni*) and KH327/KH134 (*P* = 0.026, *S. sintoni*). However, this analytical test was only significant between KH493 and KH108 (*S. dentata* and *S. antennata*) for the *Cyt b* mitochondrial gene within the subgenus *Sergentomyia*.

**Fig. 4:**
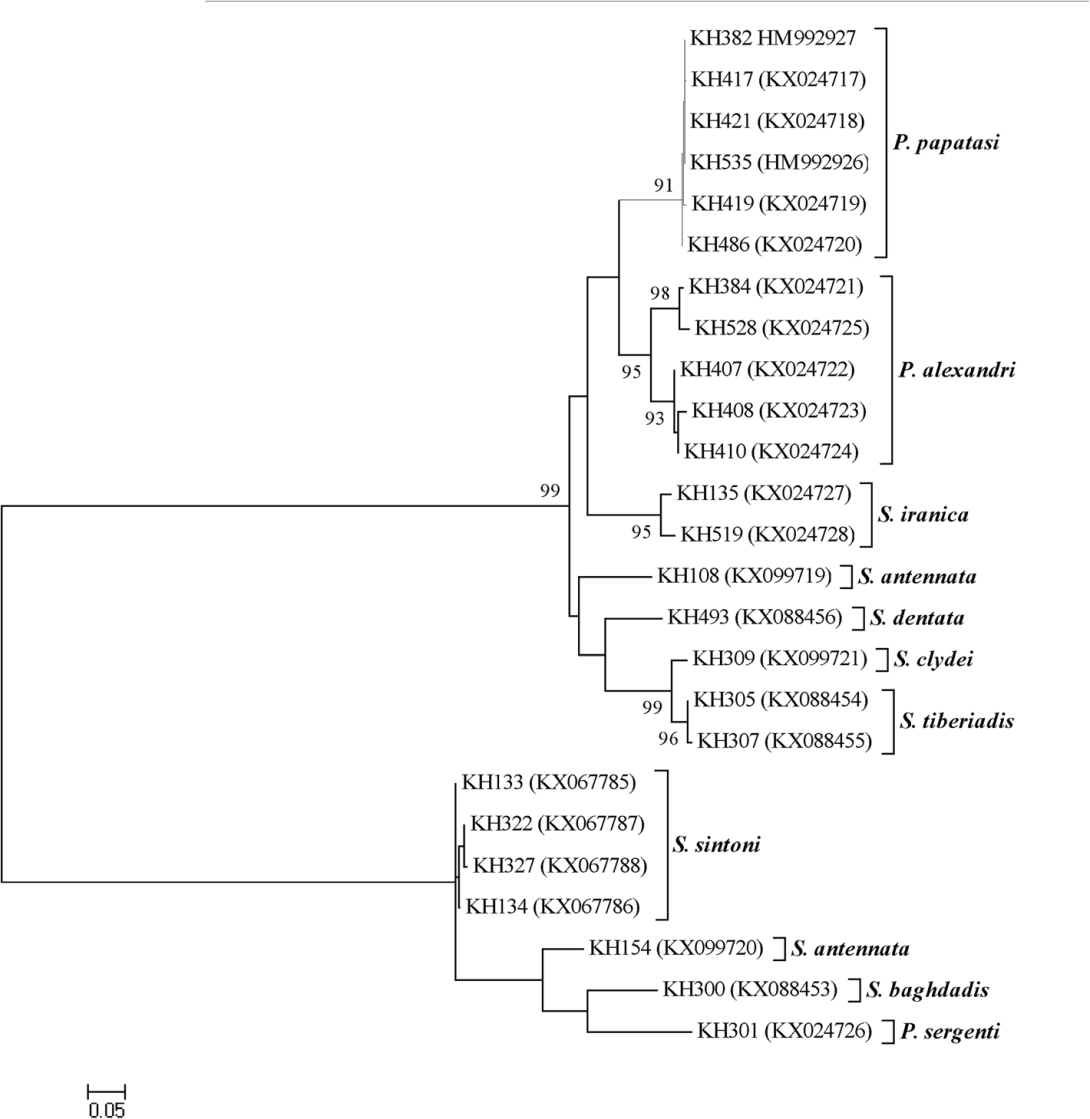
The phylogeny of the species of *Phlebotomus* and *Sergentomyia* based on maximum-likelihood (ML); the tree was constructed based on a multiple sequence alignment (haplotypes) of the mitochondrial *Cyt b* gene. The tree with the highest log likelihood (-5765.5028) is shown. Only bootstrap values higher than 70 % in which the associated taxa clustered together are indicated next to the branches. When the number of common sites was < 100 or less than one fourth of the total number of sites, the maximum parsimony method was used; otherwise BIONJ method with MCL distance matrix was used. Distance represents the number of base substitutions per site. The analysis involved 25 nucleotide sequences. All ambiguous positions were removed for each sequence pair. The (Ts/Tv) rate is 1.3664 among 897 sites in the final dataset

**Fig. 5:**
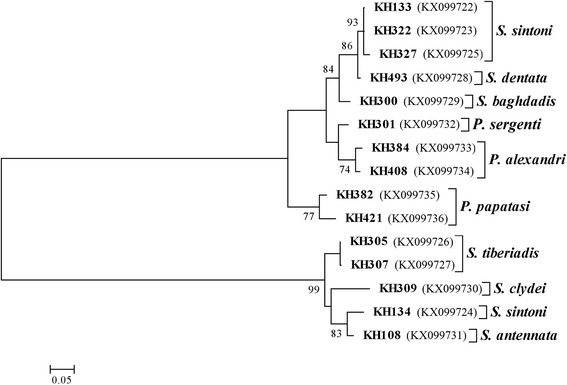
The phylogeny of the species of *Phlebotomus* and *Sergentomyia* based on maximum-likelihood (ML); the tree was constructed based on a multiple sequence alignment (haplotypes) of the nuclear *EF-1α* gene. Only bootstrap values higher than 70 % are indicated for each branch. Distance represents the number of base substitutions per site. The (Ts/Tv) rate is 1.3222 among 516 sites in the final dataset

The phylogenetic tree of approximately most caught sand flies shows similarity among different haplotypes in the related clade; however, the subgenus *Sergentomyia* was not resolved as a monophyletic clade, which is consistent with our previous findings (Figs. 4, 5) [10]. The statistical correlations were not significant between geographic distances and genetic variation based on the Mantel test (*R*^2^ = 0.3716, *P* = 0.10). Therefore, the results show that geographic distance did not have a significant effect on the genetic differentiation observed in the *Sergentomyia* and *Phlebotomus* populations.

## Discussion

Accurate identification of sand fly species based on molecular analyses is a valuable approach for determining the epidemiological aspects and distribution of natural populations in unexamined areas. In our investigation, using phylogenetic analysis, molecular characterization was performed to identify the population structure of the sand flies that were collected along the Khuzestan borders, which distinctly revealed the previously unrecorded species *P. sergenti* to be in a separate clade of the *Cyt b* region (Fig. 4). This study shows that each sand fly species includes unique sequences of the nuclear (*EF-1α*) and mitochondrial (*Cyt b*) gene markers. Certain sand fly species, such as *P. papatasi* and *P. alexandri*, featured a greater number of different haplotypes than the other sand fly species, which is related to specific geographical distances (Dashte-Azadegan, Khorramshahr and Abadan).

Finding the non-native species *P. sergenti* in new areas where no record exists implies expanding distribution of the vectors and their adaptation to local habitat conditions or environmental modifications along the Khuzestan borders (Table 1). In addition, *L. tropica* had been isolated from human ulcers at the same locations in the Dashte-Azadegan district (Susangerd and Howeyzeh) [31]. The two endemic districts of ZCL in Khuzestan Province, Khorramshahr and Shush, were studied investigating the causes of sand fly biodiversity [32] and *S. christophersi* was found as a new record. However, we could not locate *S. christophersi* from the western borders of Khuzestan Province or west of Shush city (Fig. 2), maybe Jahanifard et al. [32] found *S. christophersi* in the east, not in the west of Shush area. Moreover, the GenBank database does not contain a records for *S. dentata*, *S. iranica, S. baghdadis* and *S. tiberiadis* and a verified record of *S. antennata* sequences for the *Cyt b* gene marker.

The transition/transversion (ts/tv) rate ratios for *P. papatasi* are *k1* = 13.726 for purines and *k2* = 9.879 for pyrimidines (Table 4). Further, the overall transition/transversion bias is *R* = 4.866 within *P. papatasi*, and the overall (ts/tv) rate is lower in *P. alexandri* sequences (*R* = 1.18) for the *Cyt b* gene, perhaps due to postmutation processes from the high mutation rate of methylated cytosines to thymine in the *P. papatasi* population. However, heterogeneity manifestations exhibit a selective advantage for survival and fitness in the sand fly population sequences along the Khuzestan borders (Table 4).

The number of interpretable sequences for the *EF-1α* gene in *P. papatasi*, *P. alexandri* and the subgenus  *Sintonius* was less than three, and the quality of the remainder was insufficient to evaluate the DNA fragments after resequencing. Perhaps this phenomenon is due to the presence of multiple loci or two copies in these species as a result of gene duplication events [10]. Therefore, the sequences were excluded from statistical analyses. The estimated overall (ts/tv) bias for the subgenus *Sergentomyia* is *R* = 3.31. Tajima’s *D* value of the nuclear gene (*EF-1α*) indicated low levels of polymorphisms (*D* > +1, *P* = 1.250596) compared with the mitochondrial *Cyt b* gene (*D* > +2, *P* = 2.041410) (Table 4). This phenomenon may be due to the nature of the nuclear gene (*EF-1α*) and the low number of nucleotide substitutions between the species of a single subgenus, which are homogenized by gene conversion [15]. The *EF-1α* gene is multi copy number, this may be there are significant differences between the studied individuals and not an actual difference.

Although the data of Tajima’s *D* test for the *Cyt b* gene of *P. papatasi* (*D* > +2) revealed higher nucleotide diversity than that observed for *P. alexandri* and the genus  *Sergentomyia* (more mutations or high polymorphisms between pairs than the number of segregating sites), different haplotypes of *P. papatasi* and *P. alexandri* were positioned in their own clades with similar rates of haplotype diversity (HD: 0.933 and 1 for *P. papatasi* and *P. alexandri*, respectively) (Fig. 4). In spite of different values of Tajima’s *D*, the haplotype diversity was nearly the same for all species for both *Cyt b* and *EF-1α* genes (HD = 1). The positive Tajima’s *D* value in Khorramshahr and Abadan could be due to a relative population decrease, subdivision or recent bottlenecking among *P. papatasi*, *P. alexandri* and *Sergentomyia* spp. (Table 4). Positive values of Fu’s *F*_s_ demonstrated allele deficiency because of overdominant selection or recent population bottleneck for *P. papatasi* (Fu’s *F*_*s *_ = 3.047), *P. alexandri* (Fu’s *F*_*s *_ = 3.568) and *Sergentomyia* spp. (Fu’s *F*_*s *_ = 0.913) [33]. Based on the interpretation of tree topology in the bottleneck stage, several lineages have remained survivable under this stage without coalescing and consequently provide trees with long internal branches [33]. In fact, the topology of our phylogenetic tree obtained from the sand flies of Khuzestan borders (Figs. 4, 5) revealed the same pattern associated with the weak bottleneck stage and no effect on the fitness and survival of the sand flies. Additionally, the neutrality indices were significant for the genera *Phlebotomus* and *Sergentomyia* and this may result from selection pressure (the Z-test was *P* < 0.05 and dN/dS ratios were biased toward synonymous mutations among closely related species of sand flies) induced by environmental conditions. Pursuant to our molecular analyses, a selective sweep/hitch-hiking may have occurred in the subgenus *Sintonius* (*S. clydei* and *S. tiberiadis*) due to a population expansion or older bottlenecking, the negative values of Fu’s *F*_s_ genotype (Fu’s *F*_s_ = -0.463) indicating excess of low frequency haplotypes/alleles and beneficial mutation. We deduced that a balancing selection occurred due to a habitat modification (Iraq-Iran war). However, Tajima's *D* value of the *Cyt b* gene regions indicates an equivalent number of pairwise nucleotide differences and segregating sites (*D* = 0) for the subgenus *Sintonius*, which indicates that the population was evolving as a per mutation-drift equilibrium and therefore, increasing the fitness of the *Sintonius* spp. The observed demographic changes between the populations of *Sintonius* spp. and other sand fly species can arise from random fluctuation of neutral mutation which likely goes up or down through genetic drift and demographic events. A decrease in genetic and phenotypic variation or conservation biology (loss of biological diversity) is possible for sand fly species due to human impact [34]. Human encroachment such as habitat fragmentation, burning, logging, the presence of domestic animals, using fertilizer, natural devastation, infrastructure deterioration and civil engineering due to developments of urbanization after the Iraq-Iran war can insert large scale exchange of individuals between populations. These interventions serve as an ever increasing threat to biodiversity.

Consistent with our molecular results, we observed the rapid expansion of nucleotide differences among sequences for *Phlebotomus* spp. and the remainder of the genus *Sergentomyia* (except *Sintonius*), with considerable random mutations and low genetic variation at a geographical location along the Khuzestan borders (23 haplotypes for the *Cyt b* gene and 15 haplotypes for *EF-1α* from 108 individuals). Perhaps both, mitochondrial and nuclear genes, are involved in perfect harmony with balancing selection under imposed pressures from nature and human intervention. A GC-biased gene conversion may have occurred due to the presence of GC-rich isochores regions in the nuclear *EF-1α* gene in comparison with the mitochondrial *Cyt b* gene. However, the observed corresponding selection could be expected for housekeeping genes, as a fundamental rule underlying molecular mechanisms in establishing proper conditions for life.

Considering landscape modifications in the attacked areas after military operations of war, increased genetic homogeneity or accumulation of similar set of alleles was expected. The high rate of allele frequency and polymorphisms among the sand fly sequences found herein may be considered as an effect of genetic drift associated with habitat fragmentation and a reduction in genetic variation (extrinsic selection).

The warfare between Iraq and Iran has ruined the natural habitat of phlebotomine sand flies as anthropogenic factors [35, 36] and has introduced non-native sand flies to the Khuzestan borders. Notably, genetic tractability and later adaptation to the current ecological and environmental features have rendered conditions more propitious for vector survival. Additionally, constant change in habitat quality and differences in the flying capacities of sand fly species are the crucial factor that affects sand fly dispersal, genetic structure and population responses along the Khuzestan boundaries, where the natural habitat was lost during war.

### Phylogenetic inference based on molecular analyses

Phylogenetic analysis and molecular identification of the *EF-1α* gene revealed less genetic structuring between the *P. papatasi, P. alexandri* and *Sergentomyia* populations than for the *Cyt b* gene across the Khuzestan borders. In contrast, an empirical examination of the *Cyt b* gene indicated higher genetic variation in the populations of *P. papatasi*, *P. alexandri* and a number of species of the genus *Sergentomyia*. All closely related sand fly species, except certain species of the subgenus *Sergentomyia*, produced a higher bootstrap likelihood (> 70 %) of developing reproductive isolation (Figs. 4, 5). The constructed tree for the nuclear *EF-1α* gene produced the same results as the subgenus *Sergentomyia* for the *Cyt b* gene but showed a better clade for *Paraphlebotomus* spp. than *Cyt b*; based there on, *P. sergenti* and *P. alexandri* were grouped in a monophyletic clade (Fig. 4). Further, the specimens of KH421 and KH382, identified as unique and common haplotypes of *Cyt b* were placed in a well-supported branch of the phylogenetic tree for the nuclear *EF-1α* genotype as well. The apparent discordance in nucleotide diversity between the *Cyt b* and *EF-1α* sequences portends high variation in the maternally inherited mitochondrial gene due to elevated mutation [37]. A comparative study between the mtDNA (*Cyt b*) and nuclear gene (*EF-1α*) was performed using the phylogeny of *Larrossius* spp., but no consistent data were generated [11]. The distribution of various sand fly species, particularly *P. papatasi* and *P. alexandri,* from one location patch to another, denotes not only for physical eligibility, but also for population dynamics [38, 39] due to re-establishment of sand fly populations or wildlife corridor as a possible mitigation instead of an ever-increasing threat to biodiversity of habitat fragmentation after the 8-year Iraq-Iran war.

## Conclusion

We drew more attention to the effect of the combination of human activities and new environmental effects at the Iran-Iraq borders on vector genotypes as well as the selection and emergence of existing genotypes. The neutrality and molecular analyses suggest that our findings provide compelling evidence of randomly evolving DNA sequences from both mitochondrial and nuclear DNA haplotypes to support the fitness and survival of wild-caught sand flies with neutral mutation. Further investigation of different sand fly species of the old and new world is necessary to determine the evolution, behavior and vector competence of unknown or non-native sand fly species due to various environmental conditions and in different regions around the world, which have not yet been studied.

## Abbreviations

CIM, clustering integration method; CL, cutaneous leishmaniasis; *Cyt b*, cytochrome b; EF-1α, elongation factor 1alpha; HD, haplotype diversity; mtDNA, mitochondrial DNA; PCA, principal component analysis; ts/tv, transition/transversion; ZCL, Zoonotic Cutaneous Leishmaniasis.
